# Presence of the cloud cover and elevation angle of the sun affect measurements of eggshell coloration and patterning obtained from calibrated digital images

**DOI:** 10.1002/ece3.10170

**Published:** 2023-07-09

**Authors:** Klaudia Szala, Marcin Tobolka, Adrian Surmacki

**Affiliations:** ^1^ Department of Avian Biology and Ecology, Faculty of Biology Adam Mickiewicz University Poznań Poland; ^2^ Department of Zoology Poznań University of Life Sciences Poznań Poland; ^3^ Konrad Lorenz Institute of Ethology, University of Veterinary Medicine Vienna Vienna Austria

**Keywords:** granularity, light, normalised digital photography, repeatability, repeated‐measures ANOVA, weather conditions

## Abstract

Calibrated digital photography is frequently used in studies focusing on avian eggshell appearance to measure colour and pattern features. Photographs are often taken in natural light conditions, yet little is known to what extent the normalisation process is able to control for varied light. Here, we photographed 36 blown eggs of the Japanese quail *Coturnix japonica* at five different elevation angles of the sun on both sunny and uniformly overcast days alongside grey standards. We normalised and processed the photographs in the MICA Toolbox software and checked how much noise was introduced by different natural light conditions to the colour and pattern measurements of the same set of eggs. Our results indicate that natural variation of light conditions affects eggshell colour and pattern measurements obtained by means of calibrated digital photography. Depending on a trait, the elevation angle of the sun had similar or even greater effect on the measurement than the presence of the cloud cover. Furthermore, measurements taken in cloudy conditions were more repeatable than those taken in sunny conditions. Based on the results, we propose practical guidelines regarding measuring colour and pattern of eggshells using calibrated digital photography in outdoor conditions.

## Graphical abstract

In the article, we present how the variation of natural light conditions due to elevation angle of the sun and presence of cloud cover affect color and pattern measurements of eggshells taken with normalised digital photography. We provide practical guidelines how to cope with this issue.

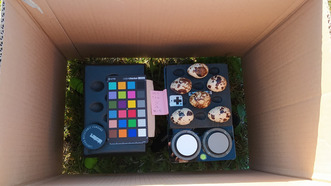

## 1 INTRODUCTION

Over the past few decades, there has been a growing interest in the pigmentation of avian eggshells. Many functions have been proposed to explain the vast diversity of this feature. Among them, there are camouflage (Gómez et al., 2018), thermoregulatory function (Bakken et al., 1978; Wisocki et al., 2020), arms‐race with brood‐parasites (Davies & Brooke, 1989; Øien et al., 1995), signalling in post‐reproductive sexual selection (Moreno & Osorno, 2003), structural reinforcement (Gosler et al., 2005) and protection of an embryo against UV light (Maurer et al., 2011).

Regardless of the hypothesis being tested, the first step in all studies is to accurately and objectively measure the eggshell pigmentation (Pike, 2019). Reflectance spectrometry is a golden standard in studies on avian integuments coloration and works well in species with uniformly coloured eggshells (e.g. López‐Rull et al. 2008; Moreno et al., 2006; but see Butler & Waite, 2016). Since spectrometers have its own stable light source, reflectance measurements are independent of ambient light variation. Moreover, information is provided with a very good spectral resolution, often down to 1 nm. Using spectrometry can be challenging, though, in the case of eggshells with finely mottled pattern. The most common approach in such cases is to measure the colour of spots and background separately (e.g. Duval, Cassey, Lovell, et al., 2013; Duval, Cassey, Miksík, et al., 2013; Hargitai et al., 2018) or to avoid spots at all and focus only on the background coloration (e.g. Cassey et al., 2010). However, since spectrometry consists of point measurements, it is technically very difficult to grasp the entire diversity of all aspects of complex eggshell patterning, like distribution and abundance of spots. Importantly, reconstructing patterning from point measurements is tedious, and the loss of information is high (Stevens et al., 2007). Finally, spectrometry is more suitable for laboratory studies or museum collections (Pike, 2011; Stevens et al., 2007). Nevertheless, it was successfully applied in a number of field studies (e.g. Moreno et al., 2006).

One of the most promising and convenient tools for assessing eggshell colour and pattern is digital photography. Fast development of this technique and growing accessibility of excellent cameras provide new opportunities and a good complement to spectrometry. Photography enables measuring the entire eggshell pattern and does not require excessive equipment, and measurement is relatively quick, which is of great importance in the case of studies in situ (Stevens et al., 2007). However, it comes with a cost of much lower spectral resolution than in the case of spectrometry, since information is usually provided only in three broad channels of a camera. To capture coloration within a human‐visible light spectrum, one needs a camera capable of producing RAW images, with manual control of exposure settings, and a set of grey standards (Troscianko & Stevens, 2015a, 2015b). Along with the development of photographic equipment, computer software designed for processing digital images and easy measurements of different aspects of eggshell appearance emerged, for instance: ImageJ with MICA Toolbox (Troscianko & Stevens, 2015a) and QCPA (van den Berg et al., 2019), SpotEgg (Gómez & Liñán‐Cembrano, 2017) and NaturePatternMatch (Stoddard et al., 2014). Such software provide a graphical user interface that facilitate linearisation and normalisation processes and output various colour and pattern descriptors (for details of normalisation and linearisation, see for example, Johnsen, 2016; Stevens et al., 2007). Additionally, programs often contain special functions dedicated to measuring eggshells' characteristics, such as fitting egg shape in MICA Toolbox (Troscianko, 2014). Another software, *pavo*, makes it possible to combine measurements from digital images and spectrometry (Maia et al., 2019).

Despite numerous advantages, it is important to bear several limitations of the photographic method in mind. While the normalisation process can cope, to some extent, with the variability of illumination, the variability of the light during the day, both in terms of intensity and the shape of spectrum, is high (Cronin et al., 2014). The presence of clouds and their thickness makes these differences even greater (Condit & Grum, 1964). Every method has its measurement error and digital photographs are no exception. It has already been outlined in the literature that corrections for a non‐linear response of a camera and for inconstant light conditions have to be made using grey standards of known reflectance (Stevens, 2011; Stevens et al., 2007). Furthermore, Troscianko and Stevens (2015b and in their extensive online User Guide http://www.empiricalimaging.com/knowledge‐base/, last accessed 8^th^ May 2023) advised taking pictures under similar weather conditions whenever possible (i.e. either sunny or overcast sky, not both) and to put effort to match the angle of the measured surface to the angle of grey standard(s), especially under directional light. In some other studies carried out in outdoor conditions, authors have also restricted the timeframe for taking pictures to make sure illumination is comparable. Mostly, they avoided early morning and late evening hours when the sun is low over the horizon (Gómez et al., 2018; Stevens et al., 2017; Troscianko, Wilson‐Aggarwal, Spottiswoode, & Stevens, 2016; Troscianko, Wilson‐Aggarwal, Stevens, & Spottiswoode, 2016; Wilson‐Aggarwal et al., 2016; but see Stoddard et al., 2016). Despite limitations and pitfalls of using digital photography to study egg coloration have been carefully discussed in the aforementioned articles, still many studies lack basic details regarding light conditions and camera settings what limits their usefulness for meta‐analyses or comparative studies.

In light of the above, this is surprising that there has been virtually no study investigating how consistent are measurements taken with digital imaging under varied natural light conditions. The exception is the project devoted to the colour of skin patches in Gelada *Theropithecus gelada* (Bergman & Beehner, 2008; DeLacey et al., 2022). However, the measurements were performed on red squares from the ColorChecker chart, which substituted skin patches. Bergman and Beehner (2008) found that measurements of colour were significantly different in overcast conditions compared to control (that was the mean of all measurements in four light conditions: sun, shaded from the sun, backlit and overcast) for two analysed X‐Rite ColorChecker red squares. Furthermore, for one of them (‘moderate red square’), measurements differed depending on whether they were taken in the morning compared to the midday and the evening. Similarly, DeLacey et al. (2022) took photographs in four different light conditions. They also used two red patches from X‐Rite ColorChecker as a substitute of the monkey's skin but converted normalised digital images into cone‐catch values of the *Papio* using MICA Toolbox software. Then, based on long and medium wavelengths, they calculated red‐green opponency as a measure of redness. Backlit conditions differed from the intercept (cloudy conditions) for ‘light skin square’, and shade and sunny conditions differed from the intercept for ‘moderate red square’.

Eggs, unlike flat skin patches, are three‐dimensional objects and may have complex patterning. Due to their spatial nature, edges of eggs are often darker in digital images (Gómez & Liñán‐Cembrano, 2017), while taking pictures in direct light can lead to glare (Troscianko & Stevens, 2015b). Moreover, eggs with pale background and dark patterning are especially difficult to measure, as it is easy either to overexpose the background or to underexpose the spots. All this makes egg a challenging object to measure its colour and pattern properly. Furthermore, two different approaches are used, depending on a tested hypothesis. In the first approach, objective colour and pattern descriptors independent of any particular receiver are used as a proxy of eggshells' pigment content. It is applied in studies dedicated to mechanical functions of pigmentation, such as, for example, structural reinforcement (Gosler et al., 2005) or protection of an embryo against ultraviolet radiation (Maurer et al., 2011). Another approach relies on visual models of an appropriate receiver and is applied in studies investigating signalling functions of eggshell appearance. In the latter case, ecologically relevant illumination conditions should be used.

In this study, we explored the constancy of colour and pattern measurements of eggshells taken under sunny and overcast weather and during different times of the day using calibrated digital photographs. We focused on the objective approach that do not assume any signal's receiver. Our aim was to work out practical guidelines regarding optimal conditions at which photographs should be taken. Such information would be of great importance for designing fieldwork plans in studies dedicated to the appearance of eggshells. Additionally, to obtain a broader picture of light conditions that are commonly used when measuring eggshell appearance by means of digital photography, we reviewed a set of research articles.

## 2 MATERIALS AND METHODS

### 2.1 Preparations and measurements

We used 36 fresh eggs of the Japanese quail *Coturnix japonica* collected on 10 April 2021 at a commercial farm in Western Poland. We selected Japanese quail eggs due to their easy accessibility and complex patterning (Duval, Cassey, Miksík, et al., 2013), which was a subject of numerous studies (e.g. Coleman & McNabb, 1975; Duval et al., 2014; Gómez et al., 2016). Eggshells of Japanese quail contain biliverdin, which is responsible for greenish hues of background coloration and protoporphyrin that give rise to brownish‐yellow background coloration and forms pattern of dark spots of variable sizes (Duval, Cassey, Lovell, et al., 2013). Since all eggs were collected on the same morning, we assume that each was laid by a different female and that samples in our study are independent. It is crucial because, as Duval, Cassey, Lovell, et al. (2013) have shown, eggshell traits of eggs laid by one female are repeatable (i.e. more affected by female identity than by environmental factors). Eggs were emptied with a syringe, cleaned with distilled water and kept in the dark before and between measurement sessions, as contact with direct light can lead to pigment degradation (Cassey et al., 2012).

The outdoor photographs were taken in an open area near the Faculty of Biology, Adam Mickiewicz University, Poznań, Poland (coordinates: N 52°28′2″, E 16°55′36″). We took pictures at five different elevation angles of the sun (the height of the sun above the horizon in degrees: 10°, 20°, 30°, 40° and 55°) in two different weather conditions: a clear blue sky without any cloud cover and uniformly overcast sky. The elevation angle of the sun and respective time of day were computed using an online calculator (https://darekk.com/sun/solar‐position‐calculator, last accessed: 15^th^ July 2021). Direct sunlight could lead to the over‐exposition of grey standards and eggs, as well as to the appearance of glare. Therefore, we photographed eggs in our own shade following other studies (e.g. Troscianko, Wilson‐Aggarwal, Spottiswoode, & Stevens, 2016; Troscianko, Wilson‐Aggarwal, Stevens, & Spottiswoode, 2016; Wilson‐Aggarwal et al., 2016). We took photographs between May and July (Table 1). This is the time period when many avian species of the northern hemisphere's temperate zone lay eggs.

**TABLE 1:** Timing of photographs series. All photographs were taken in Poznań, Poland (N 52°28′2″, E 16°55′36″). 55° is the maximum elevation angle of the sun in May at this latitude. In the case of cloudy conditions series at 10°, 20° and 30°, we took photographs in the middle of July, because rain and strong wind usually accompanied overcast sky and made it impossible to take photographs earlier.

Elevation angle of the sun (in degrees above the horizon)	Sunny conditions	Cloudy conditions
10	11.05.2021, 6:20	15.07.2021, 6:10
20	10.05.2021, 7:29	15.07.2021, 7:20
30	10.05.2021, 8:35	15.07.2021, 8:25
40	10.05.2021, 9:44	14.05.2021, 9:32 and 17.07.2021, 9:30
55	12.05.2021, 12:48	14.05.2021, 12:48

**FIGURE 1:**
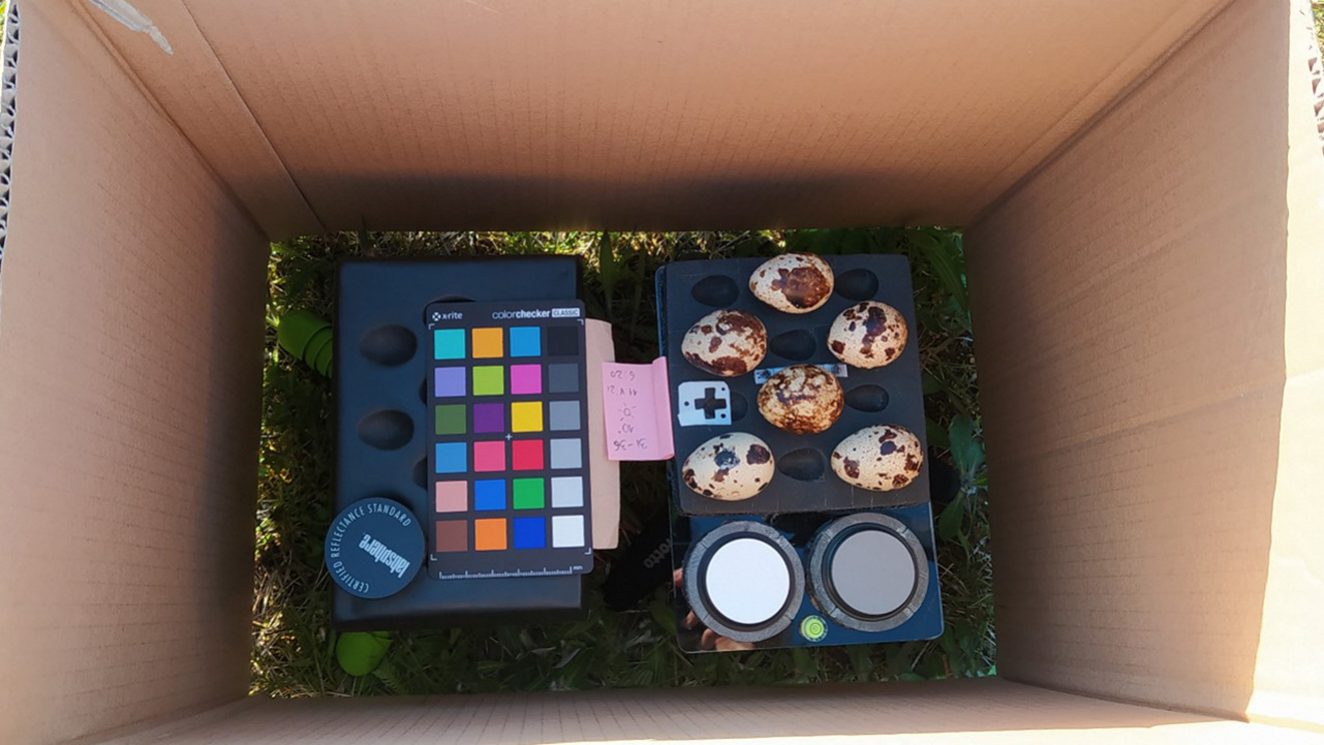
Set for photographing eggs: custom‐made supporter on a tripod with pits for eggs, grey standards and scale bar. All set was surrounded on the sides by a box to prevent Japanese quail *Coturnix japonica* blown eggs from shaking due to wind.

We used Nikon D90 converted to full spectrum with Nikkor EL 80 mm lens and photographed with Baader IR/UV cut filter, therefore, in the human‐visible light (400–700 nm). The camera was fixed to a tripod with a lens axis aligned perpendicularly to the ground. The distance between eggshells and the lens was constant (1 m). To minimise movements of the camera, we used a remote shutter release. As grey standards, we used X‐Rite ColorChecker (X‐Rite, Grand Rapids) with a scale bar present next to the eggs in every photograph. We used aperture f/8 and ISO 400 and to avoid overexposure we manipulated only the shutter speed. We used exposure bracketing with ±1 step. Depending on the ambient light intensity, integration time in our set of pictures varied between 1/1250 and 1/160 s. All images were saved as RAW files. After taking every photograph, we checked the histogram for exposure. Eggshells were put sideways on a custom‐made black plastic supporter with shallow pits for eggs (Figure 1). The supporter was aligned parallelly to the ground using a bubble level. The entire set was always surrounded by a brownish‐grey box to prevent eggs from moving by the wind.

Eggs were positioned according to the marks drawn on the eggshell. Thus, on each occasion, the same side of egg was photographed. Nevertheless, to make sure that eggs were set in the same position between consecutive pictures series, we took additional measurements of a subset of 18 eggs. We photographed them twice in artificial light in a darkroom. We used the Iwasaki eyeColor arc lamp MT70D E27 6500K (Iwasaki Electric Co., Ltd.), which has a broad spectrum of light similar to CIE D65 daylight illumination (Troscianko & Stevens, 2015b). We diffused the light with a 0.5 mm PTFE sheet that screened the eggs to avoid the glare and used the same camera setting as in the outdoor conditions.

### 2.2 Image analyses

We processed photographs in MICA Toolbox (version 2.2.1), a plug‐in for ImageJ software (Troscianko & Stevens, 2015a). Prior to colour and pattern measurements, we checked all images for exposition using the Photo Screening tool. We selected the brightest and simultaneously not over‐exposed photograph from every bracketing series. Next, in every image we selected two X‐Rite grey standard patches, the brightest and the darkest (92.96% and 1.99%, respectively, as measured with a spectrometer) to normalise it. Then, we set the scale (10 mm bar) and selected eggs using a multipoint tool to indicate eight anchor points along an egg's edges (Troscianko, 2014). We used the Scale Bar Calculator tool to check the minimum px/mm factor in our set of pictures (15.83 px/mm rounded down to 15.5 px/mm). This factor is essential in pattern analysis and should always be rounded down to rescale all images and make pattern measurements from different images comparable (Troscianko & Stevens, 2015b). Furthermore, we applied the Batch Multispectral Image Analysis tool for automatic measurements of colour and pattern. We rescaled our images and selected green channel for pattern analysis, as it closely matches the sensitivity of double cones in birds (Spottiswoode & Stevens, 2010). In pattern granularity analysis, we used the following settings: method—fast Fourier transform (FFT), start size—2 px, end size—256 px, step size—square root of 2, and steps were multiplied.

The software exports the results as a csv file where reflectance in every channel is measured compared to the reflectance of grey standards, therefore in the range between 0 (black) and 100 (white). Following methods used for spectrometry, we calculated brightness as the sum of reflectance in red, green and blue channels, and red chroma as reflectance in red channel divided by brightness (e.g. Butler & Waite, 2016; Hargitai et al., 2016). Further, following DeLacey et al. (2022), we converted our images into human cone‐catch in CIE D65 illumination and calculated red‐green opponency as (LW − MW)/(LW + MW). We also selected *sumPower* from granularity analysis as our measure of total pattern contrast, *maxPower* as a measure of contrast for dominating spot size, *propPower* as a measure of pattern diversity and *maxFreq* as a measure of dominating spot size (Troscianko & Stevens, 2015b). Granularity analysis based on fast Fourier bandpass filtering is often used to describe patterns of animals or eggshells (e.g. Stoddard et al., 2019; Šulc et al., 2019).

### 2.3 Statistical analyses

All statistical analyses were performed in RStudio (version 4.0.5, R Core Team, 2021). To prepare diagnostic plots and plots with results, we used the following packages: *ggplot2* (Wickham, 2016), *ggsignif* (Ahlmann‐Eltze & Patil, 2021), *ggpubr* (Kassambara, 2021).

To check if measurements of the same eggs taken in different light conditions differ significantly, we used repeated‐measures ANOVA with two independent variables: weather and elevation angle of the sun, as well as their interaction. The weather was a factor with two levels (‘sun’ and ‘cloud’), while the elevation angle of the sun was a factor with five levels (10°, 20°, 30°, 40° and 55°). We checked the assumptions and calculated repeated‐measures ANOVA in *rstatix* package (Kassambara, 2021, see Appendix S1 for details). Generalised eta square (*ges*, hereafter) measures how much variation is explained by each model term and is a recommended effect‐size statistic for repeated‐measures designs (Bakeman, 2005), thus, we used it in our study. Pairwise comparisons were calculated using the Bonferroni correction for multiple comparisons.

Granularity's *maxFreq* is a variable corresponding to the size of dominating spot size on a logarithmic scale. Therefore, prior to analyses, we applied log2 transformation to obtain the values on a linear scale, from 1 (the smallest spot size) to 8 (the biggest spot size) in 0.5 increments. Since this is a discrete variable, we could not use repeated‐measures ANOVA and used the Friedman test instead. Because it is a one‐way test, we aggregated our two variables: weather and degrees into a one‐factor variable, ‘level’, a combination of these two (cloud10, sun40 etc.). To perform Friedman test, we used *rstatix* package (Kassambara, 2021).

To estimate the repeatability of measurements taken in different light conditions, we calculated the inter‐correlation coefficient using the LMM‐basing approach for Gaussian data in *rptR* package (Nakagawa & Schielzeth, 2010; Stoffel et al., 2017) and following the same transformations as for ANOVA analysis (see Appendix S1). We calculated repeatability for the whole dataset (‘natural’), as well as separately for sun and cloud conditions. Additionally, we calculated the repeatability of measurements for a subset of 18 eggs photographed in constant artificial light conditions to check how much noise arises from putting the eggs on the egg‐holder and selecting their edges in the software. In all cases, we used 500 bootstraps to obtain 95% confidence limits. We did not calculate repeatability for *maxFreq* variable due to its discrete character. Data and R code used for the statistical analyses are available from the Zenodo Digital Repository (Szala et al., 2023) and from the GitHub repository.

### 2.4 Literature review

To investigate how other authors approached the issue of variable light in their research, we reviewed the literature as follows. We looked for all papers that cited the work of Troscianko and Stevens (2015a) in Google Scholar (last accessed on 21^st^ October 2022). We found 334 records and filtered them by looking for the word ‘egg’ within the text, which limited the number of papers to 126. Among them, basing on abstracts and/or methods section, we looked for articles that were original research papers focused on avian eggs and used calibrated digital photography to measure eggshell colour and/or pattern trait(s). We found 22 such articles (Table S1). Furthermore, we extracted information about the conditions in which pictures were taken. We particularly focused on the time of the day, weather (i.e. overcast or clear sky) and light diffusion techniques (i.e. light diffuser or shade).

## 3 RESULTS

Results of the repeated‐measures ANOVA showed that both weather and elevation angle of the sun, as well as their interaction significantly affected all six measurements of the eggshells appearance that we focused on (Tables 2 and 3). In the case of red chroma and human red‐green opponency, variability caused by the elevation angle of the sun was higher than variability caused by the weather, as *ges* indicated. For the rest of variables *ges* of both factors and their interaction had similar values. The trait that turned out to be the most resistant to variability due to different illumination was *propPower*.

**TABLE 2:** Results of repeated‐measures ANOVA for chromatic and achromatic features for a set of 36 Japanese quail *Coturnix japonica* eggs photographed in different weather conditions and elevation angles of the sun (‘degrees’). Generalised eta square (*ges*) is the amount of variability due to the within‐subjects factor.

Effect	DFn	DFd	*F*‐value	*p*‐value	*ges*
Brightness					
Weather	1	35	160.08	<.001	0.061
Degrees	2.95	103.21	74.064	<.001	0.061
Interaction weather:degrees	2.77	96.94	99.491	<.001	0.082
**Red chroma**					
Weather	1	35	6.604	<.05	0.003
Degrees	2.67	93.37	44.609	<.001	0.017
Interaction weather:degrees	2.55	89.09	18.746	<.001	0.01
**Human red‐green opponency**					
Weather	1	35	25.123	<.001	0.018
Degrees	2.64	92.3	49.894	<.001	0.042
Interaction weather:degrees	2.73	95.72	32.099	<.001	0.041

**TABLE 3:** Results of repeated‐measures ANOVA for granularity analysis traits for a set of 36 Japanese quail *Coturnix japonica* eggs photographed in different weather conditions and elevation angles of the sun (‘degrees’). Generalised eta square (*ges*) is the amount of variability due to the within‐subjects factor.

Effect	DFn	DFd	*F*‐value	*p*‐value	*ges*	
sumPower						
Weather	1	34	259.303	<.001	0.2	
Degrees	2.51	85.21	104.067	<.001	0.183	
Interaction weather:degrees	2.36	80.3	114.3	<.001	0.211	
**maxPower**						
Weather	1	34	282.972	<.001	0.129	
Degrees	2.7	91.68	78.23	<.001	0.092	
Interaction weather:degrees	2.32	78.99	79.294	<.001	0.111	
**propPower**						
Weather	1	34	9.168	<.01	0.004	
Degrees	4	136	6.308	<.001	0.007	
Interaction weather:degrees	3.09	105.22	2.767	<.05	0.004	

**FIGURE 2:**
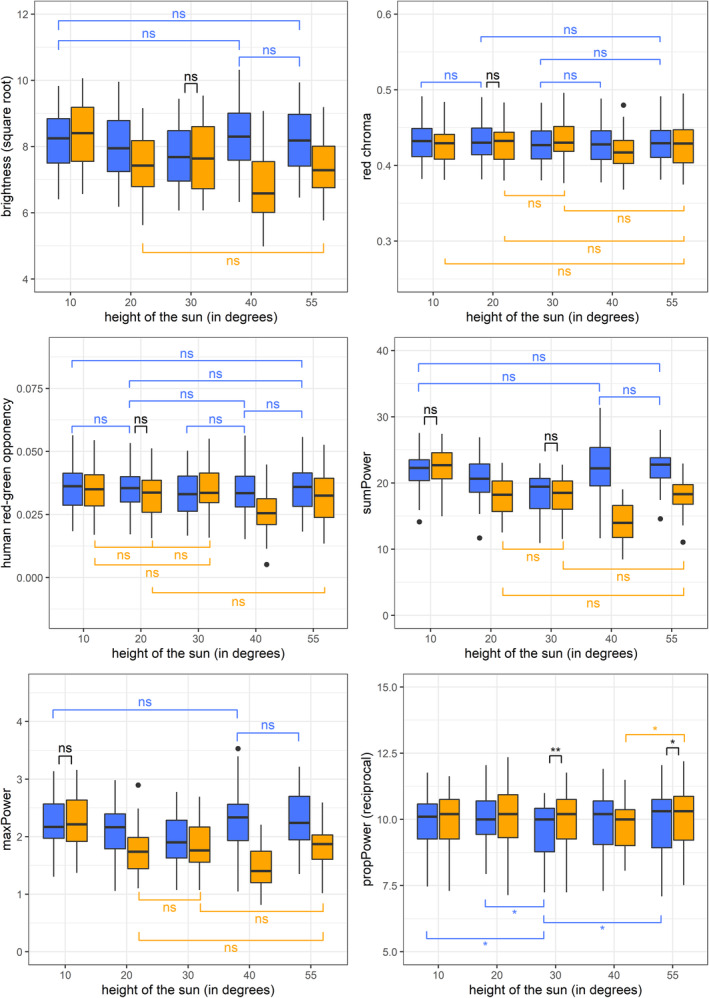
Results of the repeated‐measures ANOVA. The same set of 36 Japanese quail *Coturnix japonica* eggs was photographed in sunny (orange boxes) and cloudy (blue boxes) conditions at five different elevation angles of the sun above the horizon. For brightness, red chroma, human red‐green opponency, *sumPower* and *maxPower*, non‐significant pairwise comparisons are marked. Blue lines above boxplots connect pairs of measurements taken in cloudy conditions; orange lines pairs of measurements taken in sunny conditions; and black lines pairs of measurements taken in different weather conditions but at the same elevation angles of the sun. In the case of *propPower*, significant pairs are shown for clarity (there were too many non‐significant comparisons), and the meaning of the colour is the same as for the rest of the plots, *p < .05; **p < .01.

Since the interaction between weather and the elevation angle of the sun was significant in all cases, we additionally calculated one‐way ANOVA models where we checked the effect of the elevation angle of the sun on either level of the weather factor (Table 4). In the case of *propPower*, the effect of the elevation angle of the sun was similar for both weather conditions, whereas, for the rest of variables, the effect of the elevation angle of the sun was more pronounced in the sunny conditions. In Figure 2, we presented all pairwise comparisons that did not significantly differ from each other (except for *propPower*, where significantly different pairs were marked because there were too many non‐significantly different comparisons for the plot to be clear and interpretable). Apart from *propPower*, red chroma and human red‐green opponency were the traits that had the most non‐significant pairs of measurements—which is also indicated by their low values of *ges* (Table 2). Also, for every trait, there were from one to three pairs of measurements taken under different weather conditions, but at the same elevation angle of the sun, that did not differ significantly (Figure 2).

**TABLE 4:** Effect of the elevation angle of the sun (‘degrees’) at each level of the weather variable for a set of 36 Japanese quail *Coturnix japonica* eggs photographed in different weather conditions and elevation angles of the sun. *p*‐value was adjusted with Bonferroni correction. Generalised eta square (*ges*) is the amount of variability due to the within‐subjects factor.

Weather	Effect	DFn	DFd	*F‐value*	*p*.adj	*ges*
Brightness						
Cloud	Degrees	2.38	83.38	23.866	<.001	0.04
Sun	Degrees	2.88	100.8	143.846	<.001	0.204
**Red chroma**						
Cloud	Degrees	2.55	89.13	12.59	<.001	0.003
Sun	Degrees	2.66	93.2	32.276	<.001	0.05
**Human red‐green opponency**						
Cloud	Degrees	2.9	101.37	7.832	<.001	0.008
Sun	Degrees	2.69	94.08	47.907	<.001	0.14
**sumPower**						
Cloud	Degrees	2.07	70.29	37.722	<.001	0.168
Sun	Degrees	2.7	91.7	268.136	<.001	0.47
**maxPower**						
Cloud	Degrees	2.13	72.32	29.143	<.001	0.078
Sun	Degrees	2.53	86.13	146.127	<.001	0.292
**propPower**						
Cloud	Degrees	4	136	4.447	<.01	0.011
Sun	Degrees	2.84	96.67	4.167	<.05	0.01

**FIGURE 3:**
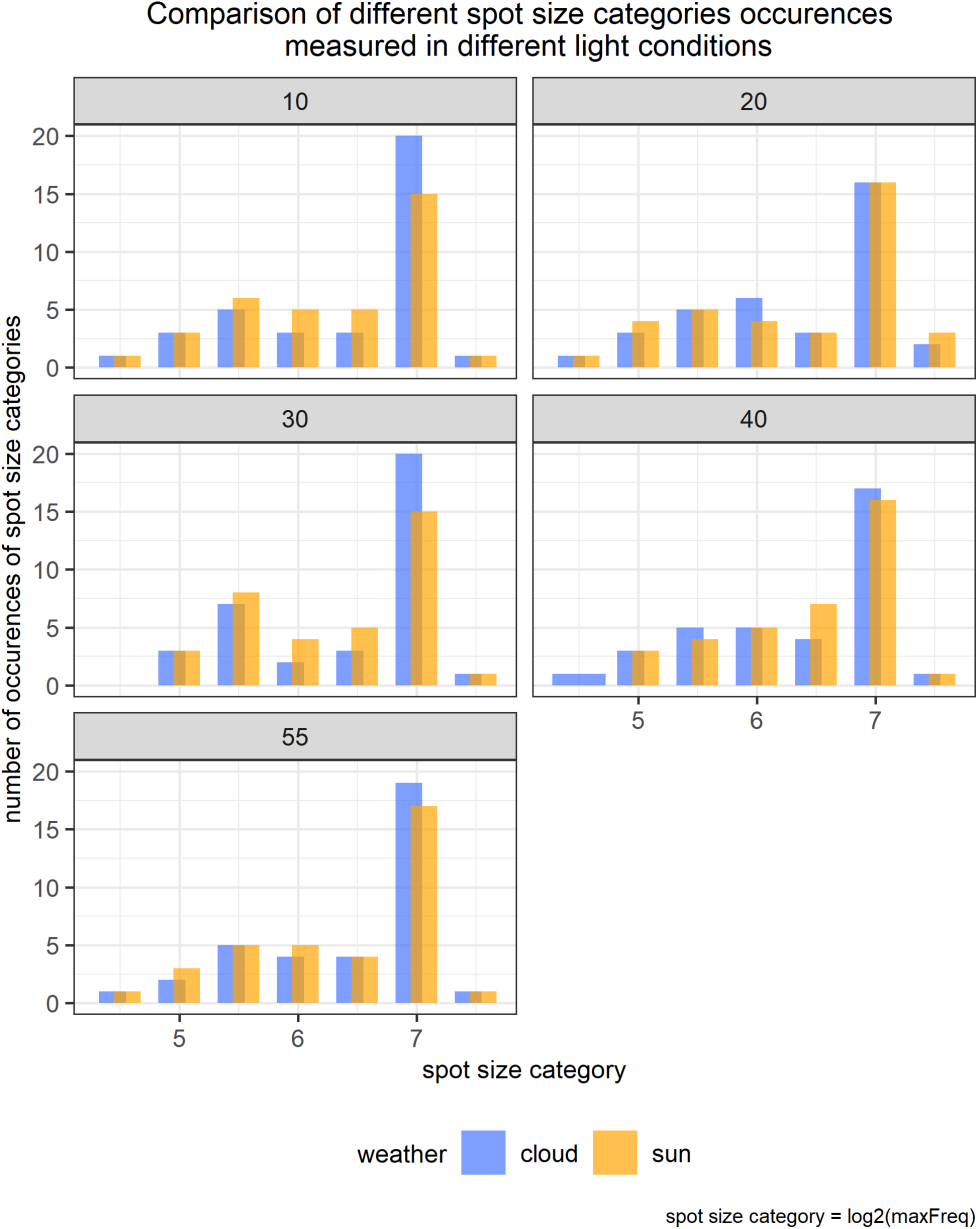
Histograms with comparison of occurrences of spot size categories in different light conditions. The same set of 36 Japanese quail *Coturnix japonica* eggs was photographed in sunny and cloudy conditions at five different elevation angles of the sun. Numbers above facets indicate elevation angle of the sun above the horizon (in degrees), and different weather conditions are expressed as colour. Spot size category is *maxFreq*, value from the granularity analysis after log2 transformation to acquire linear scale of spot size categories.

The Friedman test revealed no significant effect of illumination on *maxFreq* values (Friedman *χ* ^2^ = 6.7013, df = 9, *p* = .6682), and the additional test showed that the effect size was small according to Cohen's interpretation guidelines (Kendall's *W* = 0.0207). Figure 3 presents a histogram of occurrences of different spot size categories in different light conditions.

**FIGURE 4:**
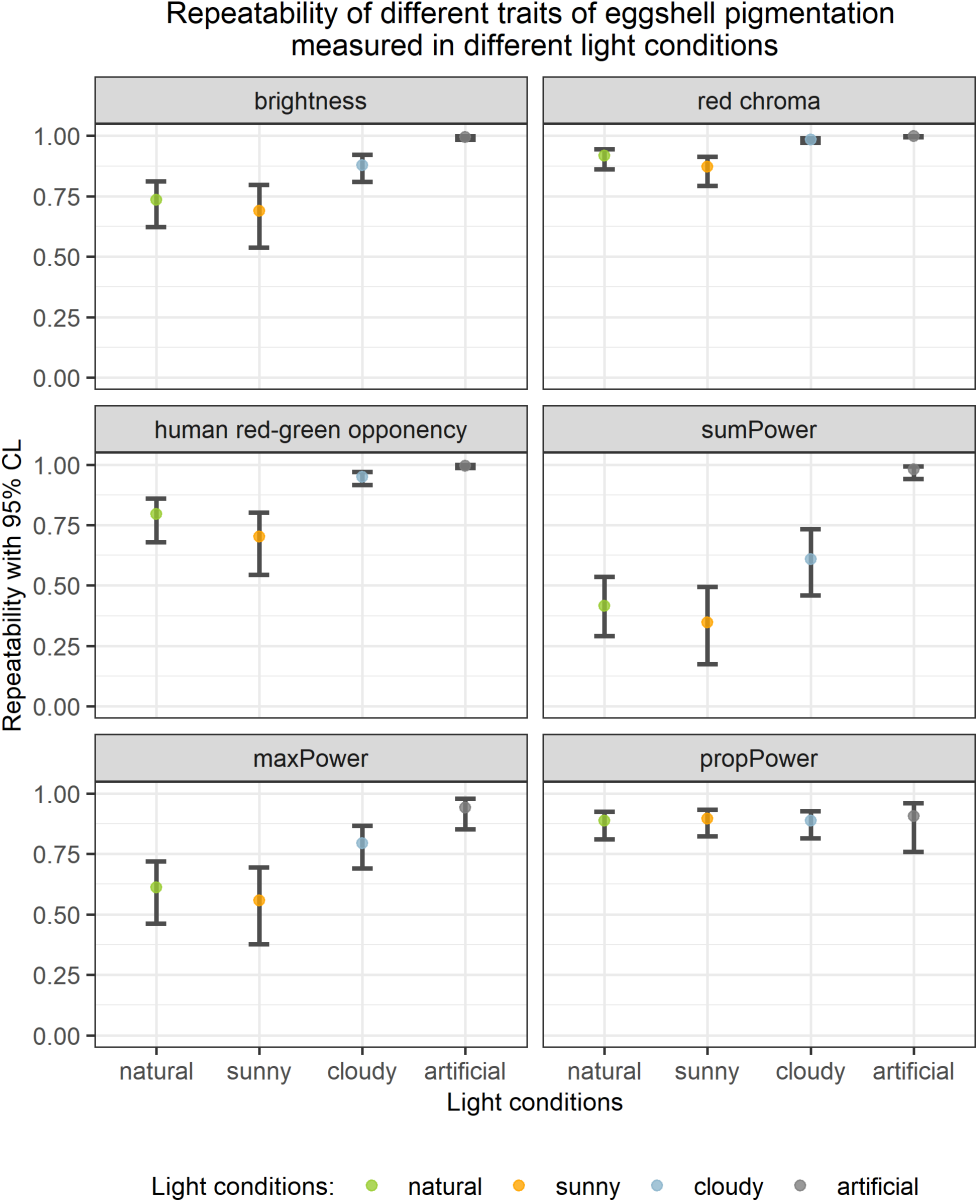
LMM‐based repeatability and 95% confidence limits of measurements of six eggshells' colour and pattern traits. The same set of 36 Japanese quail *Coturnix japonica* eggs was photographed in sunny and cloudy conditions at five different elevations angles of the sun. Category ‘natural’ is a combination of sunny and cloudy categories. Additionally, a subset of 18 eggs was photographed twice in constant artificial light conditions (‘artificial’ category) to measure the noise that arose from putting the eggs on the egg‐holder and selecting the eggs' edges on the picture.

Depending on the eggshell colour or pattern feature, the repeatability of measurements highly varied (Figure 4; Table S2). It was the lowest for *sumPower* measured in sunny conditions (*R* = 0.347, 95% CI: 0.175–0.494) and the highest for red chroma measured in cloudy conditions (*R* = 0.984, 95% CI: 0.971–0.990). In fact, the repeatability of red chroma measured in cloudy conditions in different elevations of the sun was almost as high as repeatability of red chroma measured twice in constant artificial light. Except for *propPower*, in all cases, the repeatability of measurements taken in cloudy conditions was higher than the repeatability of measurements taken in sunny conditions or than repeatability calculated for the entire dataset (combined data for sunny and cloudy conditions). Moreover, for brightness, red chroma and human red‐green opponency, 95% confidence intervals of repeatability at both weather conditions did not overlap. Instead, for *propPower*, 95% confidence intervals of repeatability of measurements taken in both weather conditions highly overlapped, with repeatability for sunny conditions being slightly higher (Figure 4; Table S2).

In constant artificial light, all measured traits had repeatability higher than 0.9, the highest for red chroma (*R* = 0.998, 95% CI: 0.995–0.999) and the lowest for *propPower* (*R* = 0.907, 95% CI: 0.759–0.959; Table S2).

In 13 out of 22 reviewed articles, pictures were taken in natural light. Among these, in five cases photographs were taken only under sunny conditions, whereas in the remaining eight the authors did not provide details about the weather. There were no studies where pictures were taken only under cloudy conditions. In next three cases, artificial light was used for taking photographs, and in the remaining six papers, no information was provided on whether pictures were taken under natural or artificial light. Furthermore, among 13 studies that photographed in natural light conditions, only in 6 articles authors restricted their measurements to selected parts of the day (and thus, to certain elevation angles of the sun). Mostly, they avoided early morning and late evening hours when the sun is low above the horizon. Finally, in three studies, photographs were taken in direct light; in six, the authors used a diffuser to diffuse light; and in four, the authors took pictures in the shade. In the remaining nine cases, there was no information on whether the light was direct or diffused (Table S1).

Validation using X‐Rite's four grey patches and red, green and blue patches (see Appendix S1 for details) showed a similar pattern of change as for the eggs (Tables S3 and S4).

## 4 DISCUSSION

The light intensity and spectrum are highly variable in the course of a day and due to weather (Condit & Grum, 1964; Cronin et al., 2014), and our results show that the normalisation process is not fully capable to cope with such differences. This confirms that measurements should be taken in uniform weather conditions, as Troscianko and Stevens (2015b) advised. Furthermore, we show that there is another, often overlooked, factor that affects the measurements and that cannot be fully controlled for by the normalisation, namely the elevation angle of the sun. Our analysis indicated that the elevation angle of the sun and weather had a similar effect on brightness and granularity analysis traits. Instead, in the case of red chroma and human red‐green opponency the elevation angle of the sun affected the measurements even to a greater degree than the weather.

The excellent repeatability (nearly 1.0) of measurements taken in artificial light clearly shows that we managed to minimise error due to the arrangement of eggs on the egg‐holder. Thus, we always measured the same side of every egg and accurately selected eggs' edges in the MICA Toolbox software. Hence, the ambient light was the only factor that accounted for differences between our measurement series. However, to prevent eggs from shaking due to wind, we always surrounded our setup with a brownish‐grey cardboard box (Figure 1). Even though the box was rather dull in coloration, it could contribute to some extent to smaller consistency of measurements in red channel comparing with, for example, green channel (Table S4; Figures S2 and S3). We also did not control for the colour of our clothes when taking photographs. However, since we photographed eggs in our shade, we were always turned with the back to the sun. Therefore, no direct light could reflect from our clothes and reach the eggs.

Behind the normalisation process, there lay assumptions that grey standards are positioned at the same distance as the measured object and that the angle between the light source and the sample, as well as between the light source and the grey standards, match (Troscianko & Stevens, 2015b). While it is possible to align standards and flat objects such as for example skin patches (Bergman & Beehner, 2008; DeLacey et al., 2022), it is impossible to do so with a tri‐dimensional object such as egg, and thus, this assumption will never be met. The curvature of the eggs results in a non‐uniform reflection of light, depending on where a point is localised on the egg surface: the closer the point is to the visible edge of an egg, the reflection is lower (Gómez & Liñán‐Cembrano, 2017).

It is worth underlining here that, except *propPower*, measurements of all analysed traits were more repeatable when sky was overcast compared to sunny conditions. In particular, the repeatability of red chroma under cloudy conditions was almost as high as repeatability of measurements from pictures taken in artificial light conditions (Figure 4). This is consistent with the ANOVA results that also show the variance introduced by the elevation of sun was lower in the cloudy conditions. It is important to note that we were not able to control for the cloud layer thickness. One could expect that this variable would introduce some noise into data and that sunny conditions would be more uniform, but our results showed the opposite. Cloud cover diffuses light, compensating for the eggs' curvature and making eggs more uniformly illuminated. It is essential to underline that all photographs in the study were taken in a shade. Thus, the results of our analysis indicate that shading eggs is not enough to diffuse light properly in sunny conditions. Therefore, we recommend taking photographs when the sky is overcast than when it is clear. Another option is to photograph during sunny weather using light diffusers, but unfortunately, we did not include such light conditions in our study design.

Alternatively, a box with an artificial light source was successfully applied in some studies (e.g. Poláček, Griggio, et al., 2017; Poláček, Bartíková, & Hoi, 2017; Ornés et al., 2014; Rahn & Ornés, 2020). Such an approach allows to omit the issue of ambient light variation. However, there are two other pitfalls: the light source should have a broad and flat spectrum that resembles the spectrum of sunlight (Troscianko & Stevens, 2015b) and light should be diffused to avoid glare, over‐exposition and, again, illuminate the eggs uniformly.

As the sun goes down, its radiance decreases and shifts to longer wavelengths (Johnsen, 2012). Therefore, we expected the measurement taken at 10° in sunny conditions to stand out. However, only in the case of brightness and two contrast measures (*sumPower* and *maxPower*) this measurement significantly differed from all the other four measurements taken in sunny conditions. For the rest of the traits, at least one pair of measurements did not differ, for example, 10° and 55° for red chroma (Figure 2). It may be explained by the fact that the most extreme changes in illumination occur once the sun is less than 10° above the horizon, whereas we took photographs when the sun was already 10° above the horizon. Also, since we shaded the eggs from direct sunlight, they were illuminated by the skylight. Skylight, which is scattered sunlight, is shifted to shorter wavelengths (Johnsen, 2012). However, when the cloud cover was present, it diffused not only the skylight but also sunlight that indirectly reached the eggs. Consequently, more light (including longer wavelengths) reflected from the eggs and eggs were more evenly illuminated. This is why brightness and red chroma tended to be higher in cloudy conditions (Figure 2).

The effect of inconstant natural light conditions on colour measures obtained with photography was also found in the studies on Gelada's skin patch (Bergman & Beehner, 2008; DeLacey et al., 2022). The results of Bergman and Beehner (2008) showed that measurements taken under cloudy conditions stood out and that time of day (and thus, the elevation angle of the sun) affected the red‐green ratio that they used in the study as a measure of colour. Similarly, DeLacey et al. (2022) showed that the measurements taken in different illumination (sun, shade, backlit and cloud) significantly differed, even though the images were transformed into a cone‐catch model of the *Papio*, and the measured object was a flat skin patch of uniform colour.

For most of the analysed traits, measurements did not significantly differ between photographs taken when the sun was at 40° and 55° above the horizon in overcast conditions (see pairwise comparisons in Figure 2). Therefore, we recommend this timeframe for photographing, as it minimises the noise arising from inconstant natural light conditions. At moderate geographical latitudes of the northern hemisphere in May, this is around 3 h before and after noon, which gives a reasonable amount of time for fieldwork. The higher sun is, the more uniform illumination and the less shade it provides. Thus, measurements taken at elevation angles above 55° would probably bring similar results, but we encourage researchers from lower latitudes to test this idea.

The trait that was highly affected by the light conditions was *sumPower*, especially in sunny conditions. The variability due to weather and elevation of the sun was almost as high as the variability in our sample of eggs for this trait (Figure S4). Therefore, it should be used with caution if the light varies between measurements, especially when pictures are taken in sunny weather. On the other side, red chroma measured in cloudy conditions had very high repeatability and seemed quite resistant to the changes in illumination. Similarly, the Friedman test showed that light conditions had a minor effect on the spot size measurement. However, there is another point that one has to bear in mind when selecting a colour or pattern trait for analyses: whether and how this trait correlates with the content of the pigment in the eggshells of the studied species. The more a colour or a pattern trait correlates with pigment content or concentration, the better (e.g. Gómez et al., 2019; Wegmann et al., 2015, but see Butler & Waite, 2016). Such an approach is often used in studies on mechanical and structural functions of eggshell pigmentation, as it does not assume any specific observer and seeks to objectively measure the amount of the pigments. Alternatively, in the studies on signalling functions of the eggs' appearance, one should transform the image into an appropriate visual model of a signal receiver, for example, a predator for camouflage function and a host species for anti‐brood‐parasite function. In such a case, variable light is of lower importance, and instead, one should put effort into taking photographs of eggs in conditions in which potential observer would see them (Stevens, 2011; Troscianko, Wilson‐Aggarwal, Stevens, & Spottiswoode, 2016).

To conclude, first, we would like to encourage researchers to work out a uniform protocol to align grey standards and measured object(s) when planning the fieldwork. Second, it is essential to keep in mind that the normalisation process cannot compensate for all changes in illumination. Therefore, one should limit photographing to selected weather conditions, preferably overcast, or use a light diffuser, and restrict fieldwork to a certain timeframe. Alternatively, one can use a box with artificial light source to eliminate the noise that natural lighting conditions can introduce into data. Finally, we encourage other authors to provide more details about the conditions in which photographs were taken, so results of different projects can be compared, as proposed by Kemp et al. (2015).

## AUTHOR CONTRIBUTIONS

**Klaudia Szala:** Conceptualisation (equal); data curation (lead); formal analysis (lead); funding acquisition (lead); investigation (equal); methodology (equal); visualisation (lead); writing—original draft (lead); writing—review and editing (equal). **Marcin Tobolka:** Conceptualisation (equal); formal analysis (supporting); funding acquisition (supporting); methodology (equal); supervision (equal); writing—review and editing (equal). **Adrian Surmacki:** Conceptualisation (equal); investigation (equal); methodology (equal); supervision (equal); writing—review and editing (equal).

## CONFLICT OF INTEREST STATEMENT

The authors declare no conflict of interest.

## Supporting information


Appendix S1:

## Data Availability

Data available from the GitHub repository (https://github.com/KlaudiaSzala/eggshell‐photography‐variable‐light) and archived on Zenodo (Szala et al., 2023, https://doi.org/10.5281/zenodo.7976182).
