# Supplementary material for: Presence of the cloud cover and elevation angle of the sun affect measurements of eggshell coloration and patterning obtained from calibrated digital images
Source: Ecol Evol. 2023 Jul 9;13(7):e10170. doi: 10.1002/ece3.10170 (PMC10329936; doi:10.1002/ece3.10170)
Supplement: Supplementary file 1 — Appendix S1: [file ECE3-13-e10170-s001.docx]

**Appendix 1**

**Table A1.** Review of light conditions in the original studies that used calibrated digital photographs to measure the eggshell pigmentation. “Sun” in the “light” column means that the authors photographed only in sunny conditions, while “natural” means that photos were taken in outdoor conditions with no further information about the weather. The column “part of day” presents if the authors restricted measurements to a selected part of day (and thus elevation angles of the sun). Hours are in the 24-hour time notation; NA = not applicable. The rightmost column contains information about light diffusing technique applied in the studies (direct light, shade or diffused light). We looked for all papers that cited the paper of Troscianko and Stevens (2015a) in Google Scholar (last accessed on 21.10.2022). We found 334 records and filtered them searching for the word „egg” within the text. Basing on abstracts or/and methods section, we selected articles that were original research papers focused on avian eggs and that used digital photographs to measure eggshell pigmentation trait(s).

| first author | year | title | light | part of day | direct light / diffused light / shade |
| --- | --- | --- | --- | --- | --- |
| Abernathy, V.E. | 2022 | Empirical evidence of coevolution between the channel-billed cuckoo and its host, the pied currawong | no info | no info | no info |
| Dixit, T. | 2022 | Visual complexity of egg patterns predicts egg rejection according to Weber’s law | natural | no info | shade |
| Šulc, M. | 2022 | Automatic identification of bird females using egg phenotype | sun | no info | diffused light |
| Attisano, A. | 2021 | Discrimination and ejection of eggs and nestlings by the fan-tailed gerygone from New Caledonia | no info | no info | no info |
| Birkhead, T. | 2021 | Exceptional variation in the appearance of Common Murre eggs reveals their potential as identity signals | natural | no info | direct light |
| Lund, J. | 2021 | Coevolutionary causes and consequences of high-fidelity mimicry by a specialist brood parasite | natural | no info | shade |
| Liñán-Cembrano, G. | 2021 | Quail eggs in artificial nests change their coloration when exposed to ambient conditions: implication for studies on nest predation | natural | no info | no info |
| Nahid, M.I. | 2021 | No evidence of host-specific egg mimicry in Asian koels | no info | no info | no info |
| Quach, L. | 2021 | Egg patterns as identity signals in colonial seabirds: a comparison of four alcid species | artificial | NA | diffused light |
| Abernathy, V.E. | 2020 | Empirical evidence of different egg morphs that match host eggs in the brush cuckoo (*Cacomantis variolosus*) | no info | no info | no info |
| Stoddard, M.C. | 2019 | Higher-level pattern features provide additional information to birds when recognizing and rejecting parasitic eggs | no info | no info | no info |
| Štětková | 2019 | Factors affecting response of the host towards parasitic egg | natural | no info | diffused light |
| Šulc, M. | 2019 | Mimicry cannot explain rejection type in a hostebrood parasite system | natural | no info | diffused light |
| Gómez, J. | 2018 | Individual egg camouflage is influenced by microhabitat selection and use of nest materials in ground-nesting birds | natural | between 9:00 and 11:00 | no info |
| Gómez, J. | 2018 | Latitudinal variation in biophysical characteristics of avian eggshells to cope with differential effects of solar radiation | artificial | NA | diffused light |
| Hwang , I. | 2018 | Effects of eggshell coloration on egg cannibalism among Glaucous-winged Gulls | artificial | NA | no info |
| Abernathy, V.E. | 2017 | Egg mimicry by the pacific koel: mimicry of one host facilitates exploitation of other hosts with similar egg types | no info | no info | no info |
| Stevens, M. | 2017 | Improvement of individual camouflage through background choice in ground-nesting birds | sun | two hours after sunrise – two hours before sunset | direct light |
| Stoddard, M.C. | 2016 | Camouflage and clutch survival in plovers and terns | natural | 6:30 – 9:00 | diffused light |
| Troscianko, J. | 2016 | Camouflage predicts survival in ground-nesting birds | sun | two hours after sunrise – two hours before sunset | shade |
| Troscianko, J. | 2016 | Nest covering in plovers: How modifying the visual environment influences egg camouflage | sun | one hour after sunrise – one hour before sunset | shade |
| Wilson-Aggarwal, J.K. | 2016 | Escape distance in ground-nesting birds differs with individual level of camouflage | sun | two hours after sunrise – two hours before sunset | direct light |

Literature used in the review

Abernathy, V.E., Bonds, E., Warner, H., Liang, W., 2022. Empirical evidence of coevolution between the channel-billed cuckoo and its host, the pied currawong. Biol. J. Linn. Soc. 20, 1–10. https://doi.org/10.1093/biolinnean/blac099

Abernathy, V.E., Liang, W., 2020. Empirical evidence of different egg morphs that match host eggs in the brush cuckoo (*Cacomantis variolosus*). Emu 120, 322–332. https://doi.org/10.1080/01584197.2020.1856686

Abernathy, V.E., Troscianko, J., Langmore, N.E., 2017. Egg mimicry by the pacific koel: mimicry of one host facilitates exploitation of other hosts with similar egg types. J. Avian Biol. 48, 1414–1424.

Attisano, A., Sato, N.J., Tanaka, K.D., Okahisa, Y., Ueda, K., Gula, R., Theuerkauf, J., 2021. Discrimination and ejection of eggs and nestlings by the fan-tailed gerygone from New Caledonia. Curr. Zool. 67, 653–663. https://doi.org/10.1093/cz/zoab066

Birkhead, T.R., Thompson, J.E., Cox, A.R., Montgomerie, R.D., 2021. Exceptional variation in the appearance of Common Murre eggs reveals their potential as identity signals. Ornithology 138, 1–13. https://doi.org/10.1093/ornithology/ukab049

Dixit, T., Apostol, A.L., Chen, K.C., Fulford, A.J.C., Town, C.P., Spottiswoode, C.N., 2022. Visual complexity of egg patterns predicts egg rejection according to Weber’s law. Proc. R. Soc. B Biol. Sci. 289. https://doi.org/10.1098/rspb.2022.0710

Gómez, J., Ramo, C., Troscianko, J., Stevens, M., Castro, M., Pérez-Hurtado, A., Liñán-Cembrano, Gustavo Amat, J.A., 2018. Individual egg camouflage is influenced by microhabitat selection and use of nest materials in ground-nesting birds. Behav. Ecol. Sociobiol. 72, 1–10. https://doi.org/1 0.1 007/s00265-01 8-2558-7

Gómez, J., Ramo, C., Stevens, M., Liñán-Cembrano, G., Rendón, M.A., Troscianko, J.T., Amat, J.A., 2018. Latitudinal variation in biophysical characteristics of avian eggshells to cope with differential effects of solar radiation. Ecol. Evol. 8, 8019–8029. https://doi.org/10.1002/ece3.4335

Hwang, I., 2018. Effects of Eggshell Coloration on Egg Cannibalism among Glaucous-winged Gulls. Honors Thesis, Andrews University.

Liñán-Cembrano, G., Castro, M., Amat, J.A., Perez, A., Rendón, M.Á., Ramo, C., 2021. Quail eggs in artificial nests change their coloration when exposed to ambient conditions: Implication for studies on nest predation. PeerJ 7, 1–14. https://doi.org/10.7717/peerj.11725

Lund, J., 2021. Coevolutionary causes and consequences of high-fidelity mimicry by a specialist brood parasite. MSc Thesis, University of Cape Town.

Nahid, M.I., Fossøy, F., Stokke, B.G., Abernathy, V., Begum, S., Langmore, N.E., Røskaft, E., Ranke, P.S., 2021. No evidence of host-specific egg mimicry in Asian koels. PLoS One 16, 1–22. https://doi.org/10.1371/journal.pone.0253985

Quach, L., Miller, A.E., Hogan, B.G., Stoddard, M.C., 2021. Egg patterns as identity signals in colonial seabirds: a comparison of four alcid species. J. Exp. Zool. Part B Mol. Dev. Evol. 336, 595–605. https://doi.org/10.1002/jez.b.22945

Stevens, M., Troscianko, J., Wilson-Aggarwal, J.K., Spottiswoode, C.N., 2017. Improvement of individual camouflage through background choice in ground-nesting birds. Nat. Ecol. Evol. 1, 1325–1333. https://doi.org/10.1038/s41559-017-0256-x

Stoddard, M.C., Hogan, B.G., Stevens, M., Spottiswoode, C.N., 2019. Higher-level pattern features provide additional information to birds when recognizing and rejecting parasitic eggs. Philos. Trans. R. Soc. B Biol. Sci. 374. https://doi.org/10.1098/rstb.2018.0197

Stoddard, M.C., Kupán, K., Eyster, H.N., Rojas-Abreu, W., Cruz-López, M., Serrano-Meneses, M.A., Küpper, C., 2016. Camouflage and clutch survival in plovers and terns. Sci. Rep. 6, 1–11. https://doi.org/10.1038/srep32059

Štětková, G., 2019. Factors affecting response of the host towards parasitic egg. BSc Thesis, Masarykova Univerzita [in Czech with English summary].

Šulc, M., Hughes, A.E., Troscianko, J., Štětková, G., Procházka, P., 2022. Automatic identification of bird females using egg phenotype. Zool. J. Linn. Soc. 195, 33–44. https://doi.org/10.1093/zoolinnean/zlab051

Šulc, M., Troscianko, J., Štětková, G., Hughes, A.E., Jelínek, V., Capek, M., Honza, M., 2019. Mimicry cannot explain rejection type in a host–brood parasite system. Anim. Behav. 155, 111–118. https://doi.org/10.1016/j.anbehav.2019.05.021

Troscianko, J., Wilson-Aggarwal, J., Spottiswoode, C.N., Stevens, M., 2016. Nest covering in plovers: How modifying the visual environment influences egg camouflage. Ecol. Evol. 6, 7536–7545. https://doi.org/10.1002/ece3.2494

Troscianko, J., Wilson-Aggarwal, J., Stevens, M., Spottiswoode, C.N., 2016. Camouflage predicts survival in ground-nesting birds. Sci. Rep. 6, 1–8. https://doi.org/10.1038/srep19966

Wilson-Aggarwal, J.K., Troscianko, J.T., Stevens, M., Spottiswoode, C.N., 2016. Escape distance in ground-nesting birds differs with individual level of camouflage. Am. Nat. 188, 231–239. https://doi.org/10.1086/687254

**Detailed description of the assumptions of the repeated-measures ANOVA**

To meet the assumptions of normality, we square rooted the brightness. In the case of *sumPower* and *maxPower* we removed observations of one egg (no. 7), from all cells, as it was an outlier. This egg had an additional calcium layer on the very surface of the eggshell, with small very bright dots of calcium, which may be the reason of its exceptionally high contrast value. In the case of *propPower* we found one outlier (egg no. 12 that had exceptionally big spot that covered almost all blunt end of the egg) and applied reciprocal transformation, but despite this, the distribution in two cells still differed from the normal distribution (Shapiro-Wilk test: W = 0.933 and 0.935, p = 0.034 and 0.039 respectively), and therefore results of repeated-measures ANOVA for *propPower* should be treated with caution. Other variables had normal distribution, with the exception of maxFreq, which is a discrete variable, and therefore we used Friedman test for the latter.

**Table A2.** Detailed results of repeatability analysis (inter-correlation coefficient using LMM-basing approach for Gaussian data in *rptR* package for R). Natural light conditions refers to all data set while sunny and cloudy were subset of measurements taken respectively under clear sky and uniformly overcast sky at different height of the sun above the horizon (10, 20, 30, 40 and 55 degrees). Artificial light conditions refers to a subset of 18 eggs measured twice under constant artificial light.

| light conditions | trait | repeatability | lower 95% CI | upper 95% CI | p-value |
| --- | --- | --- | --- | --- | --- |
| natural | brightness | 0.736 | 0.624 | 0.812 | <0.001 |
| sunny | brightness | 0.689 | 0.537 | 0.797 | <0.001 |
| cloudy | brightness | 0.879 | 0.81 | 0.921 | <0.001 |
| natural | red chroma | 0.918 | 0.862 | 0.945 | <0.001 |
| sunny | red chroma | 0.873 | 0.793 | 0.914 | <0.001 |
| cloudy | red chroma | 0.984 | 0.971 | 0.99 | <0.001 |
| natural | human red-green opponency | 0.795 | 0.68 | 0.861 | <0.001 |
| sunny | human red-green opponency | 0.703 | 0.545 | 0.801 | <0.001 |
| cloudy | human red-green opponency | 0.95 | 0.915 | 0.969 | <0.001 |
| natural | sumPower | 0.416 | 0.292 | 0.536 | <0.001 |
| sunny | sumPower | 0.347 | 0.175 | 0.494 | <0.001 |
| cloudy | sumPower | 0.608 | 0.46 | 0.733 | <0.001 |
| natural | maxPower | 0.612 | 0.462 | 0.719 | <0.001 |
| sunny | maxPower | 0.558 | 0.377 | 0.695 | <0.001 |
| cloudy | maxPower | 0.794 | 0.69 | 0.867 | <0.001 |
| natural | propPower | 0.887 | 0.811 | 0.925 | <0.001 |
| sunny | propPower | 0.896 | 0.822 | 0.932 | <0.001 |
| cloudy | propPower | 0.888 | 0.815 | 0.927 | <0.001 |
| artificial | brightness | 0.994 | 0.985 | 0.998 | <0.001 |
| artificial | red chroma | 0.998 | 0.995 | 0.999 | <0.001 |
| artificial | human red-green opponency | 0.995 | 0.987 | 0.998 | <0.001 |
| artificial | sumPower | 0.980 | 0.94 | 0.993 | <0.001 |
| artificial | maxPower | 0.942 | 0.853 | 0.978 | <0.001 |
| artificial | propPower | 0.907 | 0.759 | 0.959 | <0.001 |

**Validation test using X-Rite ColorChecker four grey and three colour patches**

While the brightest and the darkest X-Rite ColorChecker grey patches were used for normalisation, the remaining four grey patches, as well as three colour patches (“red”, “green” and “blue” according to producer’s names) were used for validation. X-Rite was always photographed six times in every combination of light conditions (weather x elevation angle of sun, in total 10 light conditions).

To be able to compare photographic measurements with reflectance measured with a spectrometer, we calculated reflectance as mean of pixel values in red, green and blue channels for grey patches. Table A3 and Figure A1 below present average values and standard deviation of six measurements in every light conditions of grey patches. Overall, the general pattern of changes in grey squares is similar to the one that emerged for eggs (compare Figure A1 with the pattern for brightness in Figure 2 in the manuscript). Grey standards deviated from the known reflectance values measured with a spectrometer, and while general pattern of changes was the same for all four of them, deviations were smaller for darker and higher for brighter patches (Figure A1).

Using the same approach as for eggs, we calculated brightness, red chroma and human red-green opponency for three colour X-Rite patches: “red”, “green” and “blue” (producer’s names). Results are presented in Table A4 and Figure A2. We could not calculate other metrics used for eggs (such as sumPower, maxPower, propPower, maxFreq), as they describe pattern features while X-Rite colour patches are homogenous in coloration.

Measurements for red, green and blue patches deviated from mean values and green patch had the smallest deviations, while red patch had the highest. Similarly to eggs, there was a high variability of brightness between measurements, while variability of red chroma was much lower. Green patch had the lowest deviation from mean values, while red patch had the highest (Figure 2). Unfortunately, we cannot provide spectrometric analogues to photographic measurements (as we did for grey patches), because we do not know spectral sensitivity curves of our camera.

We also compared variability (as standard deviation) observed in our sample of 36 Japanese quail eggs with variability generated by weather and by elevation angle of the sun (Figure A4). In some cases, variability generated by weather and elevation angle of the sun is only a small proportion of variability in the sample of eggs (for red chroma, propPower, maxFreq), but in other cases it is almost as high as variability in the sample (for sumPower and human red-green opponency).

**Table A3**. Comparison of reflectance values for four X-Rite ColorChecker grey patches measured as mean pixel values in red, green and blue channel in varied light conditions: uniformly overcast (“cloud”) and sunny (“sun”) weather and at five different elevation angles of the sun: 10, 20, 30, 40 and 55 degrees. Standard ID refers to the producer’s names of the patches. Bolded rows in the bottom of every section show mean values for measurements taken in all weather conditions and reflectance values measured using spectrometry.

| Light conditions | Standard ID | Mean reflectance | sd |
| --- | --- | --- | --- |
| cloud10 | neutral 8 | 63.01 | 0.42 |
| cloud20 | neutral 8 | 62.62 | 0.9 |
| cloud30 | neutral 8 | 60.33 | 0.35 |
| cloud40 | neutral 8 | 62.8 | 1.61 |
| cloud55 | neutral 8 | 62.9 | 0.56 |
| sun10 | neutral 8 | 61.59 | 0.39 |
| sun20 | neutral 8 | 60.44 | 0.57 |
| sun30 | neutral 8 | 60.44 | 0.1 |
| sun40 | neutral 8 | 59.16 | 0.66 |
| sun55 | neutral 8 | 63.45 | 0.36 |
| **average value** | **neutral 8** | **61.67** | **1.56** |
| **spectrometric measurement** | **neutral 8** | **64.51** | **-** |
| cloud10 | neutral 6.5 | 38.77 | 0.28 |
| cloud20 | neutral 6.5 | 38.23 | 1.11 |
| cloud30 | neutral 6.5 | 35.99 | 0.36 |
| cloud40 | neutral 6.5 | 38.58 | 1.77 |
| cloud55 | neutral 6.5 | 38.69 | 0.58 |
| sun10 | neutral 6.5 | 37.36 | 0.41 |
| sun20 | neutral 6.5 | 36.01 | 0.75 |
| sun30 | neutral 6.5 | 35.99 | 0.14 |
| sun40 | neutral 6.5 | 34.2 | 0.69 |
| sun55 | neutral 6.5 | 39.11 | 0.35 |
| **average value** | **neutral 6.5** | **37.29** | **1.74** |
| **spectrometric measurement** | **neutral 6.5** | **37.35** | **-** |
| cloud10 | neutral 5 | 20.2 | 0.17 |
| cloud20 | neutral 5 | 19.7 | 0.71 |
| cloud30 | neutral 5 | 18.31 | 0.25 |
| cloud40 | neutral 5 | 19.95 | 1.28 |
| cloud55 | neutral 5 | 20.18 | 0.34 |
| sun10 | neutral 5 | 19.19 | 0.27 |
| sun20 | neutral 5 | 18.24 | 0.49 |
| sun30 | neutral 5 | 18.22 | 0.12 |
| sun40 | neutral 5 | 16.79 | 0.39 |
| sun55 | neutral 5 | 20.25 | 0.18 |
| **average value** | **neutral 5** | **19.10** | **1.22** |
| **spectrometric measurement** | **neutral 5** | **19.03** | **-** |
| cloud10 | neutral 3.5 | 8.29 | 0.06 |
| cloud20 | neutral 3.5 | 8.07 | 0.34 |
| cloud30 | neutral 3.5 | 7.64 | 0.08 |
| cloud40 | neutral 3.5 | 8.22 | 0.51 |
| cloud55 | neutral 3.5 | 8.26 | 0.13 |
| sun10 | neutral 3.5 | 8.19 | 0.1 |
| sun20 | neutral 3.5 | 7.7 | 0.22 |
| sun30 | neutral 3.5 | 7.72 | 0.05 |
| sun40 | neutral 3.5 | 6.96 | 0.16 |
| sun55 | neutral 3.5 | 8.46 | 0.07 |
| **average value** | **neutral 3.5** | **7.95** | **0.48** |
| **spectrometric measurement** | **neutral 3.5** | **8.52** | **-** |


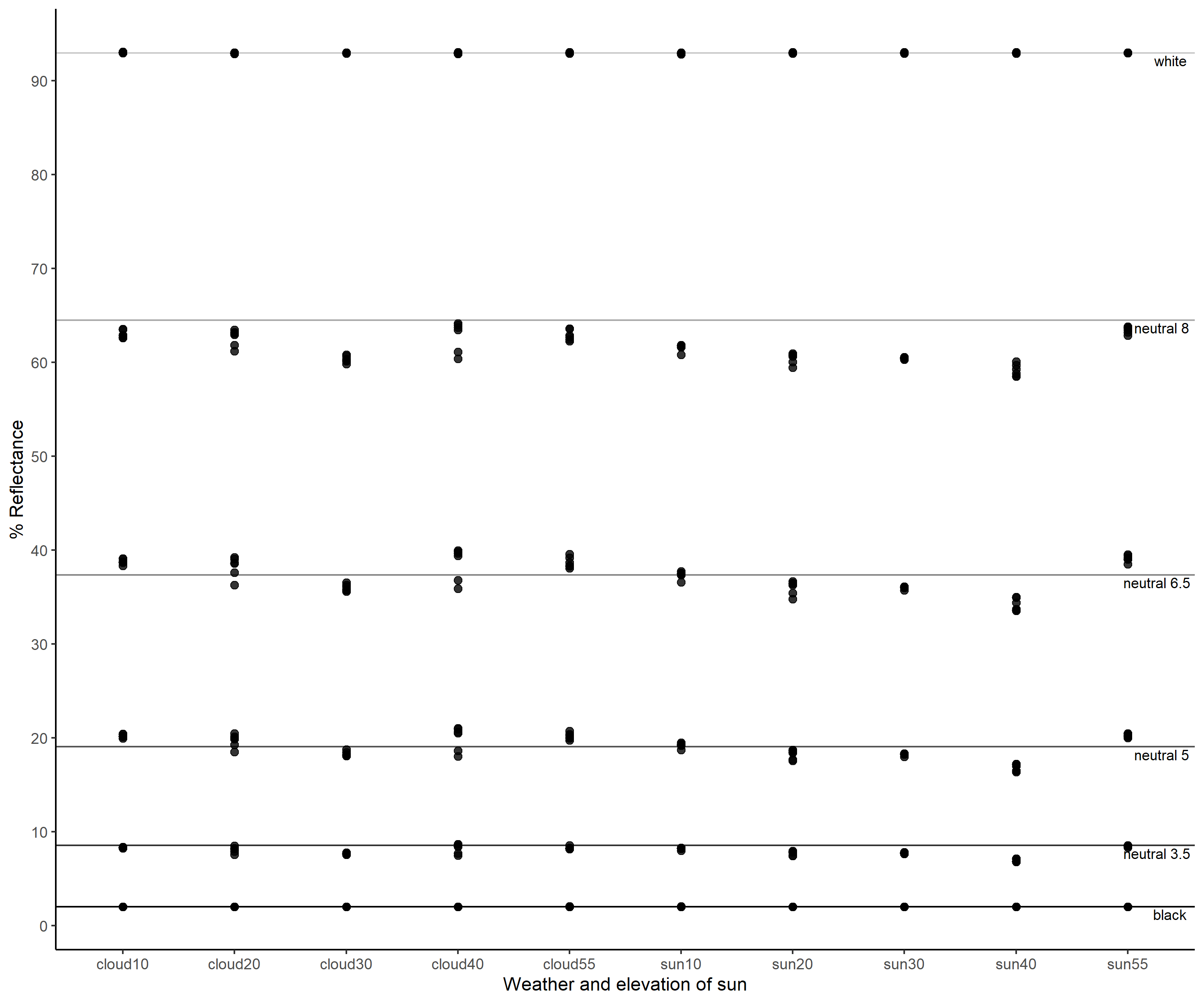


**Figure A1**. Reflectance of six grey patches of XRite ColorChecker measured by means of photography. Every dot represents reflectance measured as average pixels values in red, green and blue channel. We took six measurements in each combination of weather x elevation angle of sun (in total 60 measurements of every grey patch). Horizontal lines are reflectance for every grey patch measured using spectrometer. Top and bottom lines are for the brightest “white” and the darkest “black” grey standard respectively. These two standards were used for normalisation of the image and this is the reason why all measurements lie exactly on the line of spectrometric reflectance measurement. Names below the lines on right part of the plot are the producer’s names for grey patches.

**Table A4**. Comparison of reflectance values for three X-Rite ColorChecker patches: “red”, “green”, and “blue” (producer’s names) measured using photographic method in different lighting conditions: sunny and overcast weather and at different elevation angles of sun (10, 20, 30, 40 and 55 degrees). We calculated brightness (Br) as sum of pixel values in red, green, and blue channels, red chroma (RC) as pixel values in red channel divided by brightness and human red-green opponency (RGop) as (lw – mw) / (lw + mw). Standards were measured six times in every light conditions. Bolded rows presents average values for all light conditions for every X-Rite patch separately.

| Light conditions | standardID | Mean Br | SD Br | Mean RC | SD RC | Mean RGop | Sd RGop |
| --- | --- | --- | --- | --- | --- | --- | --- |
| cloud10 | red | 57.57 | 0.39 | 0.76 | 0.002 | 0.27 | 0.002 |
| cloud20 | red | 55.85 | 1.228 | 0.76 | 0.006 | 0.27 | 0.006 |
| cloud30 | red | 53.93 | 0.809 | 0.74 | 0.002 | 0.25 | 0.003 |
| cloud40 | red | 55.96 | 1.523 | 0.76 | 0.014 | 0.27 | 0.014 |
| cloud55 | red | 56.23 | 0.654 | 0.76 | 0.003 | 0.27 | 0.003 |
| sun10 | red | 49.66 | 0.211 | 0.76 | 0.002 | 0.27 | 0.002 |
| sun20 | red | 45.97 | 0.971 | 0.76 | 0.004 | 0.26 | 0.004 |
| sun30 | red | 46.12 | 0.34 | 0.75 | 0.001 | 0.26 | 0.001 |
| sun40 | red | 48.1 | 1.445 | 0.74 | 0.004 | 0.24 | 0.005 |
| sun55 | red | 54.45 | 0.937 | 0.77 | 0.001 | 0.28 | 0.002 |
| **average** | **red** | **52.38** | **4.36** | **0.75** | **0.011** | **0.26** | **0.012** |
| cloud10 | green | 47.11 | 0.314 | 0.24 | 0.001 | -0.05 | 0.001 |
| cloud20 | green | 46.65 | 0.495 | 0.24 | 0.002 | -0.05 | 0.001 |
| cloud30 | green | 46.5 | 0.47 | 0.24 | 0.003 | -0.05 | 0.001 |
| cloud40 | green | 46.27 | 0.9 | 0.24 | 0.006 | -0.05 | 0.002 |
| cloud55 | green | 46.09 | 0.338 | 0.23 | 0.001 | -0.05 | 0.001 |
| sun10 | green | 45.05 | 0.128 | 0.25 | 0.001 | -0.04 | 0.001 |
| sun20 | green | 44.24 | 0.191 | 0.26 | 0.001 | -0.04 | 0.001 |
| sun30 | green | 44.98 | 0.258 | 0.26 | 0.001 | -0.04 | 0.000 |
| sun40 | green | 46.17 | 0.561 | 0.25 | 0.002 | -0.05 | 0.001 |
| sun55 | green | 47.66 | 0.343 | 0.24 | 0.001 | -0.05 | 0.001 |
| **average** | **green** | **46.07** | **1.081** | **0.24** | **0.009** | **-0.05** | **0.003** |
| cloud10 | blue | 38.28 | 0.272 | 0.13 | 0.001 | -0.17 | 0.002 |
| cloud20 | blue | 38.13 | 0.69 | 0.13 | 0.003 | -0.17 | 0.007 |
| cloud30 | blue | 39.66 | 0.743 | 0.14 | 0.003 | -0.16 | 0.005 |
| cloud40 | blue | 37.06 | 2.463 | 0.13 | 0.013 | -0.18 | 0.022 |
| cloud55 | blue | 37.29 | 0.36 | 0.13 | 0.002 | -0.18 | 0.005 |
| sun10 | blue | 41.18 | 0.302 | 0.15 | 0.003 | -0.13 | 0.003 |
| sun20 | blue | 42.49 | 0.301 | 0.16 | 0.002 | -0.13 | 0.003 |
| sun30 | blue | 43.66 | 0.333 | 0.16 | 0.002 | -0.13 | 0.002 |
| sun40 | blue | 42.9 | 0.509 | 0.16 | 0.004 | -0.14 | 0.007 |
| sun55 | blue | 40.34 | 0.292 | 0.13 | 0.002 | -0.17 | 0.003 |
| **average** | **blue** | **40.10** | **2.440** | **0.14** | **0.013** | **-0.16** | **0.020** |


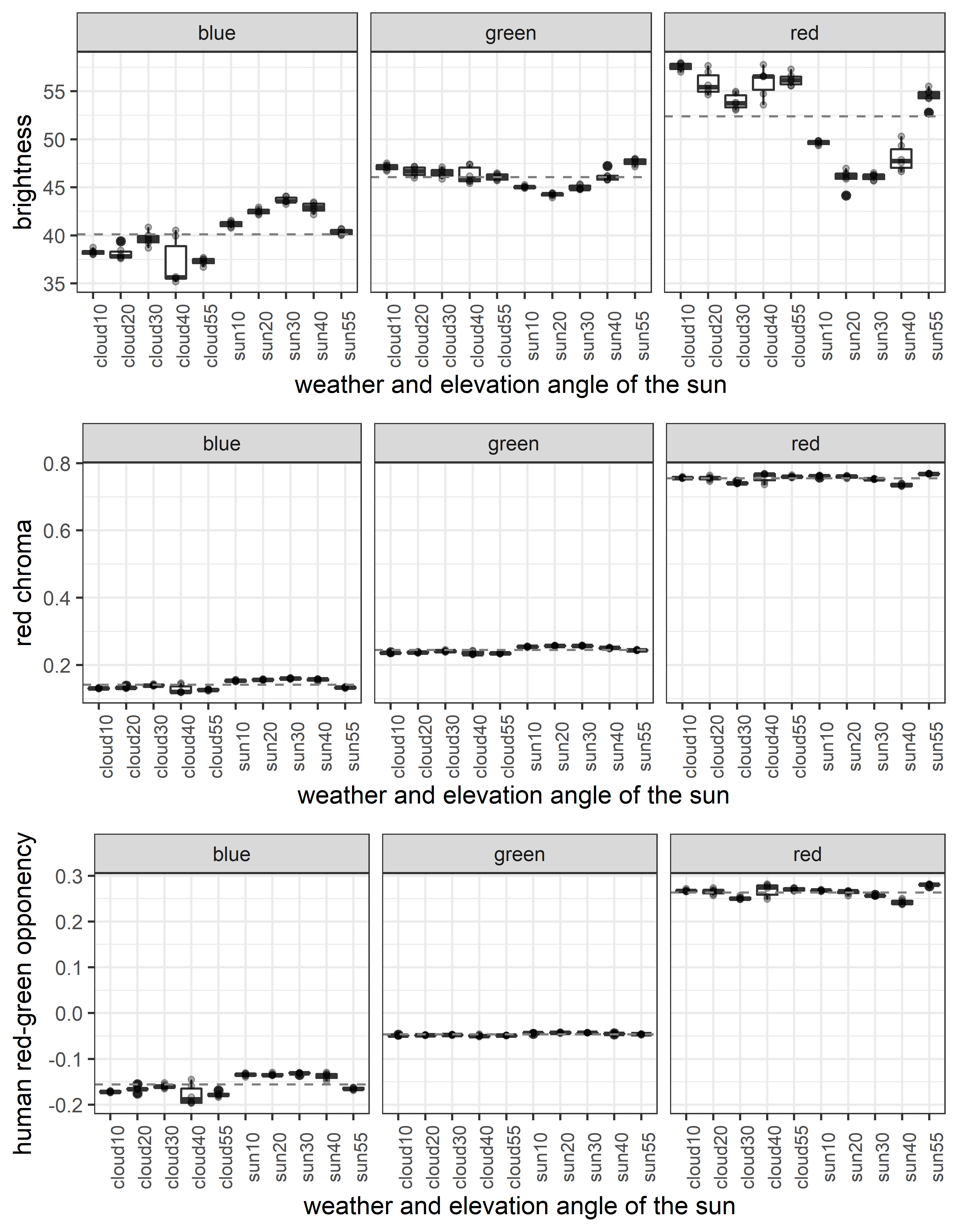


**Figure A2**. Comparison of brightness (sum of pixel values in red, green and blue channels), red chroma (pixel values in red channel divided by brightness) and human red-green opponency (lw – mw) / (lw + mw) for three X-Rite ColorChecker patches: “red”, “green” and “blue” measured using photographic method in different light conditions: sunny (“sun”) and overcast (“cloud”) weather and at different elevation angles of the sun (10, 20, 30, 40 and 55 degrees). Dashed grey lines represents mean value for all light conditions.


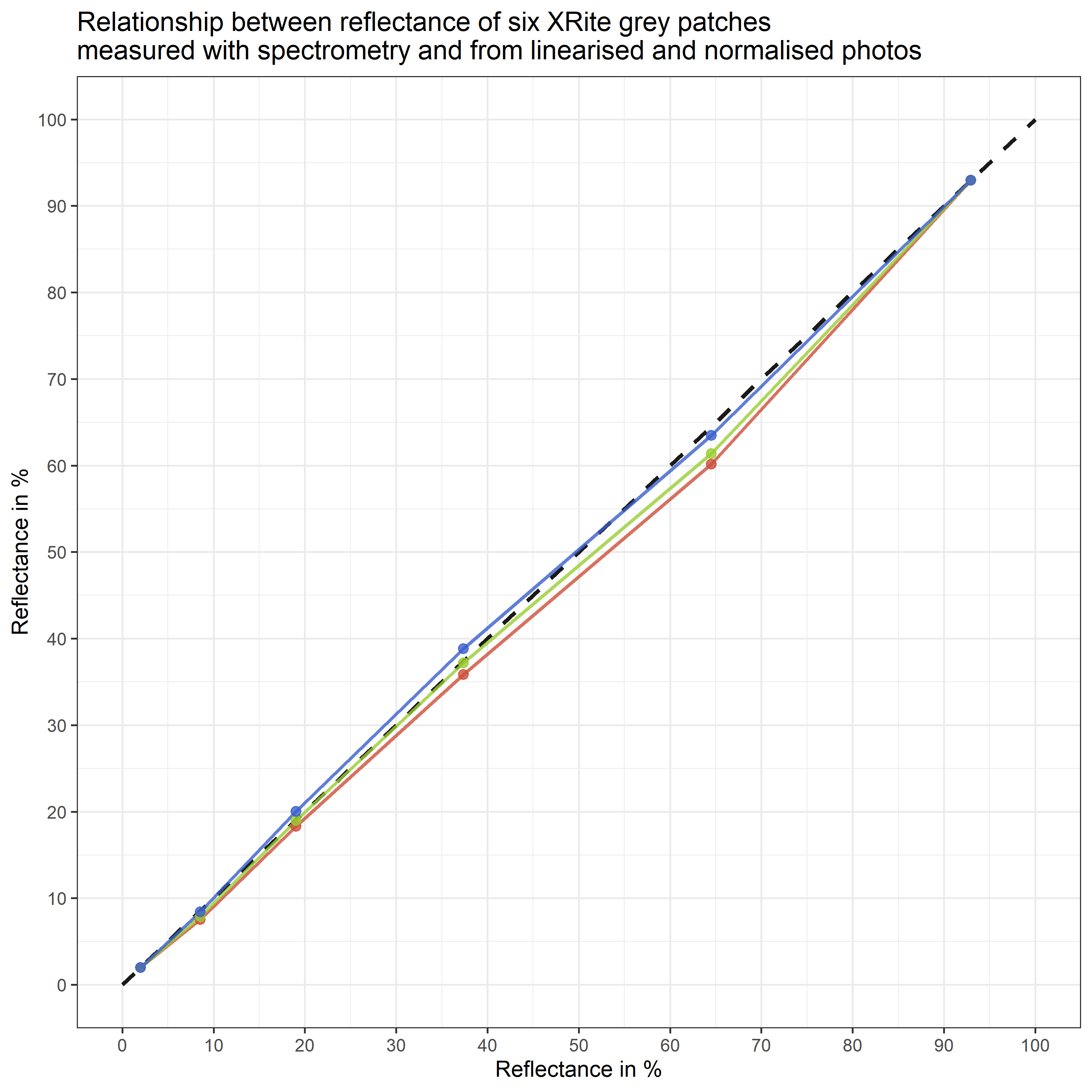


**Figure A3**. Relationship between reflectance of six X-Rite grey patches measured using spectrometry and calibrated digital photographs (mean values of all measurements). Dashed line represents linear response, while solid lines show pixel values of grey standards measured using photographic method (colours represent camera’s channels – red, green and blue respectively). The reflectance values of the darkest and the brightest lie directly on the dashed line, because these two standards were used for normalisation.


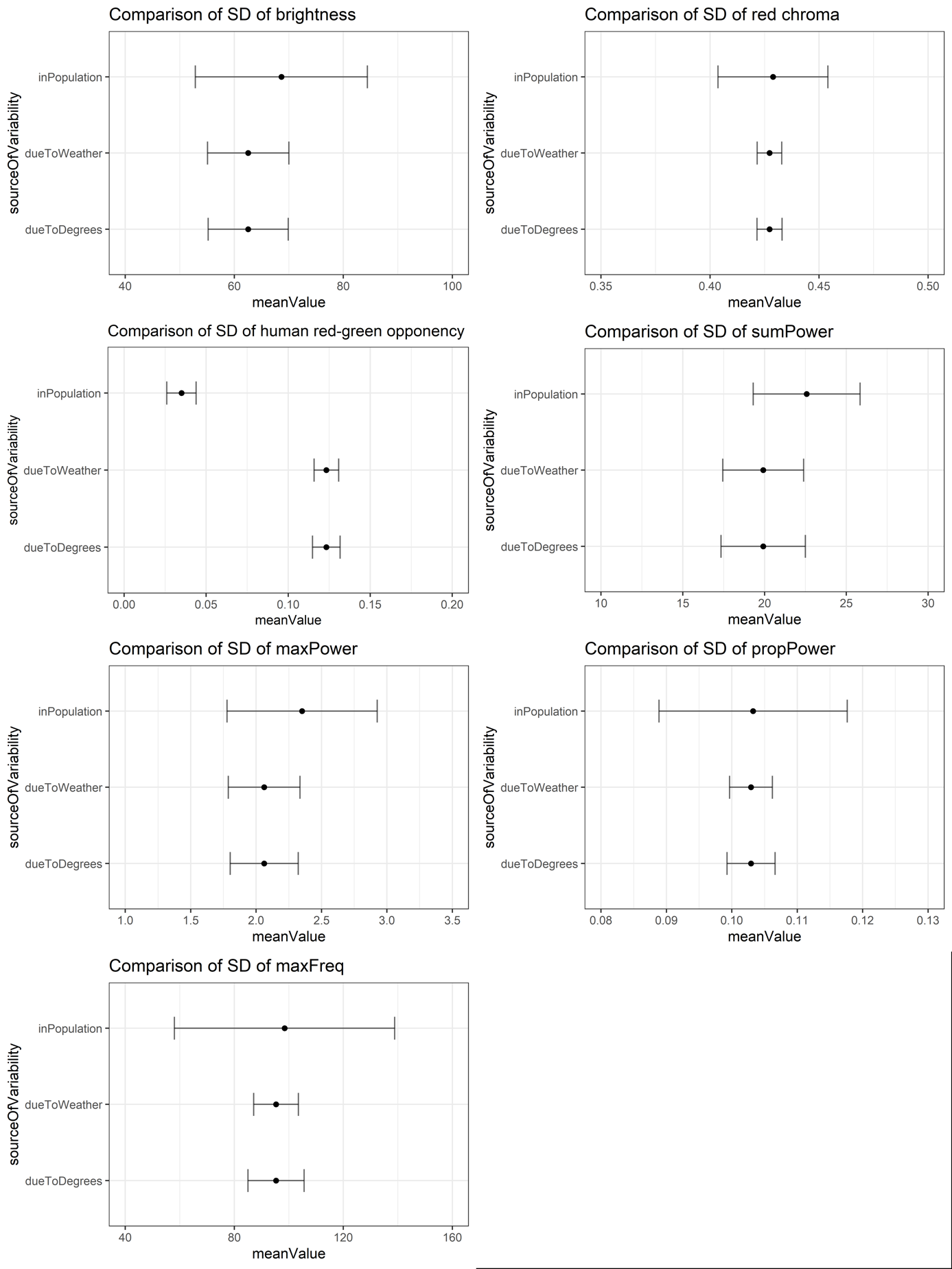


**Figure A4**. Comparison of sources of variability (standard deviation) for different traits of the eggshell pigmentation in the set of 36 eggs of Japanese quail Coturnix c. japonica photographed in different natural light condition – at different height of the sun above the horizon at both sunny and uniformly overcast day. Variability in population was calculated under cloudy conditions when the sun was 55 degrees above the horizon – this is the variability of pigmentation traits in a set of 36 eggs (every egg was from different female). Variability due to weather was mean standard deviation between two levels of weather conditions at the same level of the height of the sun. Variability due to degrees was mean standard deviation among five levels of height of the sun above the horizon at the same level of the weather. Tails represents +/- 1 SD.
